# The Human Resources for Health Program in Rwanda – Reflections on Achievements and Challenges: Comment on "Health Professional Training and Capacity Strengthening Through International Academic Partnerships: The First Five Years of the Human Resources for Health Program in Rwanda"

**DOI:** 10.15171/ijhpm.2018.114

**Published:** 2018-11-21

**Authors:** Hélène Delisle

**Affiliations:** Department of Nutrition, Faculty of Medicine, University of Montreal, Montreal, QC, Canada.

**Keywords:** Sub-Saharan Africa, Health Professionals, Capacity Building

## Abstract

This commentary is a further discussion of a paper published in this journal on the health professional training initiative led by the Government of Rwanda since 2012 and presented as a case study. According to the authors, the partnership program with international academic institutions may serve as model for other countries to address the shortage of health professionals and to strengthen institutional capacity, based on the competencybased and innovative training programs, the numbers of graduates, the improved quality of health services and institution strengthening. However, the conditions may not be as optimal elsewhere. A supportive government policy, massive funding and an academic consortium comprised of 19 United States academic institutions have contributed to the success of the program. We also noted that the trained professionals were clinicians almost exclusively, at the expense of public health specialists and other health professionals who can better address emerging issues such as non-communicable diseases (NCDs) particularly for their prevention, which is now compelling. Among others, the training of more nutritionists as members of the health team is needed.

The purpose of this commentary is to further discuss the experience of the Rwanda Human Resources for Health (HRH) program in the light of the case study published in this Journal.^1^ Some more background on medical education in Africa is also provided since the HRH program focused primarily on the training of medical professionals.

## The Evolution of Medical Education in Africa

One of the 17 Sustainable Development Goals (SDGs) for the horizon 2030 addresses health directly and several others impact indirectly on health. Towards the goal of ensuring healthy lives and promoting well-being for all at all ages, several targets were set, and skilled health professional density and distribution is among the health-related SDG indicators. In its latest progress report, World Health Organization (WHO) underlines the persistent severe shortage of qualified health professionals, with 76 countries having less than one physician per 1000 population over the period 2007-2016.^2^ Yet it has to be recognized that medical education in sub-Saharan Africa has come a long way since the colonial days.^3^ While only a handful of medical schools existed in the region in the 1960s, there were 51 in 1980 and 168 by 2009. The leading challenges to medical education scale-up were then identified as the weak infrastructure and faculty shortages. The last decade witnessed a considerable increase in funding for sub-Saharan African medical education, as it was realized at the international level that a sufficient medical workforce was needed for the battle against tuberculosis, AIDS and malaria to succeed. The US-funded Medical Education Partnership Initiative (MEPI) stemmed from the recognition of this urgent need. This 5-year project, with a budget of US$130 million, has provided support for 13 African universities, including the National University of Rwanda. The MEPI program represents a paradigm shift in medical education and North-South collaborations, as illustrated in the Rwanda case study^1^ and as also described for Mozambique.^4^ Program funds flow to the low- or middle-income country (LMIC)-based leadership group, which contracts with peers from high-income countries (HICs) to provide technical and scientific advice and consultation in a ‘reverse funds flow’ model. LMIC program partners translate broad program goals and define metrics into priorities that are tailored to local conditions. Emphasis is also placed on strengthening administrative capacity within LMIC institutions. The MEPI program is built around five core themes: (1) Strengthening training and research to enhance the capacity and quality of physicians trained; (2) Developing a critical mass of African researchers to address the most pressing health problems in their own countries; (3) Retaining health workers where they are most needed; (4) Developing communities of practice to strengthen partnerships to address common areas of interest; and (5) Achieving sustainable institutional development to ensure that MEPI accomplishments will continue beyond the initial budget period.^5^

## A Critical Look at the Rwanda Human Resources for Health Program

According to the case study report, the HRH Program may be regarded as a success story after only 5 years of operation, and it illustrates many strengths of the MEPI. The primary goal of the Program was to train a large, diverse and competent health workforce in Rwanda. This capacity strengthening program is to last 7 years (2012-2019), a consortium of more than 20 academic institutions of the United States is involved, and the initial budget from the US Government and the Global Fund was comfortable to say the least. The program was evaluated and was showing positive results even before the end of the program. It was in line with, and therefore supported by, the national development plan of Rwanda “Vision 2020,” which set as a core component the establishment of a large, diverse and competent health workforce. The program was designed to address the health workforce shortage, as well as to strengthen the capacity of the country’s health graduate schools, with investments in non-academic domains, ie, management and administration, finance, fundraising and development. Pairing of visiting and Rwandan faculty has been a promising strategy for sustainability. The program implemented competency-based training and innovative pedagogy. It also included measures for continuing education of practicing health professionals. The ownership of the program by Rwanda is another strong point. The fact that the program funds and overhead went directly to Rwanda instead of to partner US institutions with much higher overhead is positive, while also contributing to the appropriation of the program by local institutions. Although the program was in the hands of Rwanda institutions, the process for determining priorities is not entirely clear, however, and there appears to have been some struggle over this.

How the challenges posed by differences in medical training models, as well as in language and culture, were addressed is interesting and intriguing. For one, the massive support of US academic institutions was congruent with the recent shift of Rwanda from French to English as official language. It is also of note that some visiting faculty from other sub-Saharan African countries were recruited and deployed, but primarily for nursing and midwifery, in order for technical knowledge and skills to be adapted to the socio-cultural context and priorities. Cross-cultural training programs such as the HRH in Rwanda provide 2-way benefits. Rwandan faculty and students had opportunities to travel to the United States for further training and to give lectures, or to attend international conferences, all this on satellite grants. The report also mentions that the US academic institutions involved in the program derived substantial benefit through the participation of their faculty and students who could thereby learn and apply different approaches to health service delivery and even pursue careers in global health.

As stated by the authors of the report themselves, means and resources for monitoring and evaluation of the HRH Program had been underestimated. Implementation research would have been necessary to feed into the program while it was going on. Additionally, a mix of quantitative and qualitative methods would have been necessary to assess perceptions and opinions of those involved in the program. It was felt that an external ex-post evaluation would have been useful and could have also assessed program outcomes and even impact on health. However, linking improved health outcomes with training programs in health is notoriously difficult.

## Training Needs for Various Categories of Health Professionals

Although the title implied otherwise, the health professionals trained in this program were almost exclusively clinical: doctors, nurses/midwives, and dentists. The only supported program that may be connected with public health is the master of global health delivery. Additionally, the training programs that were deprioritized were family medicine and community health, as well as the advanced diploma in nursing, in order to devote the resources and expertise to medical and nursing specialty training. Yet public health competencies may have been present or integrated in the training programs, but this is not spelled out in the paper. This progressive shift toward a more clinical program is somewhat reminiscent of another university health professional training program that was developed in the Cameroon in the 1970s. The then newly established University Center for Health Sciences (UCHS) offered in its early years a very innovative training program for doctors, graduate nurses and midwives in its early years, and public health was emphasized. A faculty team including public health specialists, health educators, sociologists, nutritionists, and environmental experts was in charge of classroom and field public health training. The program developed concepts not tried before such as the problem solving approach, an integrated teaching internship training, competency-based training and team training of three different categories of health professionals.^6^ However, the program slowly drifted back to a standard medical school model focusing on clinical care and medical specialties, with the progressive slimming down of the public health component.

There is undoubtedly a paucity of clinical professionals in LMICs, as the WHO data illustrate.^2^ In 2017, the African Platform for Human Resources for Health pleaded for an enhanced role of nursing in its declaration, but surprisingly, there was no mention of other health professionals.^7^ Other categories of health professionals are needed for the health workforce to address the emerging and compelling health issues, particularly non-communicable diseases (NCDs), which are now the number-one cause of death in LMICs. Prevention is as critical as treatment, and the determinants of health have to be addressed. Whether clinicians are the best equipped and have the time to deal with these non-medical issues is questionable. Task shifting of prevention, diagnosis and treatment of NCDs to community health workers has been advocated.^8^ Community health workers may be available in higher numbers, better distributed throughout the country, and cheaper than doctors or nurses. In this realm, competencies required on the part of doctors and nurses would have to be re-defined based on the composition of the health team in the local context. WHO has provided an interesting competency framework of this sort.^9^ Of note, since 2016, the University of Western Ontario (Canada) with a long experience of medical training in Rwanda is helping to train front line health workers in order to improve care for women and young children.^10^ This has to be regarded as a useful complement to the HRH program.

The Rwanda program supported 12 new programs out of a total of 22 training programs, but it would appear that the training of nutritionists was not one of these, while malnutrition, whether undernutrition or ‘overnutrition,’ is a major public health issue in sub-Saharan Africa, particularly with the nutrition transition and the resulting rise of obesity and associated NCDs. Even in Rwanda where much progress was seen in the recent years, the prevalence of child stunting, which signals chronic malnutrition, still reached 37% in 2016.^11^ Poverty, and resulting food insecurity, is undoubtedly the root cause, but poor access to healthcare and inadequate prevention and management of acute malnutrition are also at play. Rwanda has also known the largest increase of childhood obesity of the sub-Saharan countries over the period of 1990-2012.^12^ This again underlines the importance of nutrition action to halt the progression of obesity and comorbidities. It was recognized by South African nutrition researchers that nutrition experts would need to be more aggressive in order for nutrition to be put at the forefront of the health priorities, especially in Africa,^13^ for nutrition affects virtually every public health problem. Obviously, training nutritionists is only one avenue for reducing malnutrition, as the determinants lie for most part outside the nutrition and even the health domain. Yet the critical importance of more and better trained nutritionists for improving health in Africa and for reaching the health-related SDGs has been repeatedly emphasized.^14-16^

The health workforce has to be considered as a whole and the need for interprofessional education is constantly reiterated including in WHO’s global strategy for HRH.^17^ Training in health leadership may foster the interprofessional collaboration. An inspiring experience in this regard was conducted in Uganda, where fellow doctors and nurses from several African countries were jointly trained, partly in one of the partner institutions and partly through e-learning.^18^

## The Sustainability Challenge

In spite of the substantive efforts toward this purpose, sustainability of the HRH Program will likely remain a challenge once the massive external funding comes to an end in 2019, whether considering financial, academic, professional or institutional sustainability. Policies are in place to promote the retention of the trained health workforce, including a mandatory 4 to 5-year contract of employment in the public sector for all new graduates. Interestingly, new graduates of the Master of Medicine recruited in teaching hospitals are required to devote 20% of their time to training. Continuous education programs for health professionals have also been developed in order to maintain quality of health services as well as retention of personnel. Rwandan faculty is to be recruited among the new graduates to gradually replace the visiting faculty and this process will have to be scaled up to sustain the training supported by the HRH program. This will require continued and effective twinning between visiting faculty and new Rwandan faculty. The cost cannot be overlooked: High quality training is expensive. External funding will forcibly decline gradually over time whereas the needed funds for salaries of an increasing faculty, for scholarships for a growing student body and for equipment will increase. E-learning will have to be resorted to for part of the training. Access to e-training materials in teaching hospitals was only briefly mentioned in the paper. Another issue not to be overlooked is whether public service will be able to efficiently absorb a good share of the new graduates. Additionally, the working conditions have to be acceptable if the brain-drain is to be contained.

It can be concluded that in spite of implementation and evaluation challenges, initiatives such as the HRH program can serve as examples, if not as models, for other African countries to address the shortage of qualified health professionals and for strengthening local academic institutions. The program has undoubtedly contributed to Rwanda now being considered as having one of the highest quality health systems in Africa.^19^ However, for a balanced health workforce, the training of health professionals other than doctors and nurses has to be given more emphasis. Moreover, collaboration with sectors other than health, such as agriculture, social affairs and education, should be stressed in the training of health professionals in order to address the determinants of health and to reduce health inequalities.

## Ethical issues

Not applicable.

## Competing interests

Author declares that she has no competing interests.

## Author’s contribution

HD is the single author of the paper.
